# Efficacy of linking Breast Awareness Clinics in Gynecology and Obstetrics departments: A pilot project in Zubaida Bani Wing, Fazle-Omar Hospital, Chenab Nagar, Pakistan

**DOI:** 10.12669/pjms.38.4.5440

**Published:** 2022

**Authors:** Durr-e-Samin Tahir, Wasima Tauseef, Bareah Madiha, Tahira Urooj

**Affiliations:** 1 Durr-e-Samin Tahir, Abdus Salam School of Sciences, Department of Zoology, Nusrat Jahan College, Chenab Nagar, Pakistan; 2 Wasima Tauseef Consultant Obstetrician and Gynecologist, Fazle-Omar Hospital, Chenab Nagar, Pakistan; 3 Bareah Madiha, Abdus Salam School of Sciences, Department of Zoology, Nusrat Jahan College, Chenab Nagar, Pakistan; 4 Tahira Urooj, Abdus Salam School of Sciences, Department of Zoology, Nusrat Jahan College, Chenab Nagar, Pakistan

**Keywords:** Breast Awareness Clinics, BSE, Zubaida Bani Wing, Chenab-Nagar, Pakistan

## Abstract

**Objectives:**

Pakistani female population has the highest incidence of Breast cancer among the women belonging to all other Asian countries. This contributed to 28.7% of all new cases of malignancies in 2020 and is also the cause of uppermost cancer mortality in Pakistani females. The number of deaths can be reduced by promoting breast cancer screening, according to recommended programs for high-risk populations.

**Methods:**

In order to encourage breast self-examination in women, 12 Breast Cancer Awareness Clinics were set up in Gynecology and Obstetrics outdoor, Zubaida Bani Wing, Fazle Omar Hospital, Chenab Nagar, Pakistan, from September 2019 to February 2020. A total of 238 women with a mean of 29.97±8.9 years were examined and trained to perform the breast self-examination.

**Results:**

Around 41% of 222 valid responded women reported that they had knowledge that breast cancer is the major threat to their health in Pakistan. Only 20% were aware of breast cancer screening but poor cognizance about the recommendations for Pakistani women. Knowledge about Breast Self-Examination was recorded in 15 %, whereas only 5.4 % of all the women included in the present study reported to practice it. Moreover, eighty-six percent of the respondents were reported to be hesitant to visit the male doctor. Three cases of most suspected malignancies were referred to specialist consultants and few cases were endorsed for immediate mammograms.

**Conclusion:**

Setting up breast awareness and screening clinics at gynecology outdoors with trained female assistants can be effective in promoting Breast Self-Examination and elaborating screening programs countywide to obtain long term benefits in a high-risk population of Pakistan.

## INTRODUCTION

Among the rest of Asian countries, highest incident of age adjusted breast carcinoma have been observed in Pakistani female population.^1^ Although the breast cancer (BC) incidences are greater in developed countries, relative rate of death by incidences are found to be higher in developing countries, contributing more than 60% of BC global death counts.^2^,^3^ According to the data released by Globocan in 2012, Pakistan was one of the top five countries with the highest age standardized BC mortality rate with 25.2 deaths per 100,000.^3^ Most recently, the number of new cases of BC in Pakistani female of all ages, during 2020 were recorded to be 25,928 and 13725 died by BC in this year. Five years prevalence of BC in Pakistan was reported recently to be 56.39 per hundred thousand.^4^

In Pakistan like most other Asian countries, registries for BC cases are not properly maintained at national level, thus available data about the incidence and mortality rate is considered to be insufficient.^1^,^5^ It has been reported that 89% of BC patients presents at later stages in Pakistan, mostly due to the lack of awareness;^6^ a major contributor of greater incidence to mortality ratio. In women belonging to America, it has been reported that up to 99% of the cases of BC diagnosed in the localized stages are curable, while the five-year survival rate is less than 30% among the case where cancer spread to later stages.^7^ There are many factors which are contributing to the low screening rates for BC in women from Asian countries. Some of these factors are lack of knowledge about symptoms of BC, lack of awareness about its screening, hesitation in contributing to screening programs, fear of disease, scarce financial resources, social, religious, and cultural views.^8^-^10^ The regular Breast Self-Examination (BSE) has been mentioned by various previous authors as a substantial to enable the women to notify any changes in the mammary tissues and report during clinical visits.^11^,^12^ If performed correctly and regularly, BSE is a simple, confidential, and harmless method for self-detection of breast lumps in the early stages.^13^

This study was carried out as a pilot project to set up Breast Awareness Clinics (BAC) in Obstetrics and Gynecology department of Fazle- Omar Hospital, Chenab Nagar, Pakistan, to float the idea for extending screening facility for Pakistani women. Moreover, authors aimed to increase awareness about the importance of BSE in the early detection of breast cancer, teaching the right methods of BSE and to encourage its regular practice in local female population. Another important concern was to detect and report any suspected lumps or changes in the mammary tissue of visiting females, to suggest for further follow ups by the specialists.

## METHODS

Twelve Breast Awareness Clinics were arranged in Gynecology outdoor, during September 2019 to February, 2020 in Zubaida Bani wing Fazle Omar, Hospital Chenab Nagar, Pakistan, with the approval of administration of Hospital and institutional Ethical Review Committee (NJC-196 Dated: 19-08-2019). Two hundred and thirty-eight women who were enrolled for their regular visit for pregnancy follow ups and/or gynecology problems in outdoor and the females accompanying them were advised to visit BAC, with their consent. The women were also encouraged to bring other females from their families and neighborhood for breast screening. Their breasts were examined by standard procedure of breast examination. The patients were brought into relaxed and laying down position on the stretcher. Chest region was exposed for visual inspection followed by palpations. Any changes in the skin color, size, texture, and shape of breast were observed. The nipples were observed for deformity or discharge. The breast tissue was inspected for any lumps by exerting circular gentle pressure from inwards to outwards on the breast by using pads of the three fingers of hand. The patients were advised to keep their respective arm under the head and chest tissue was examined up to the armpit. Most of the women were asked to perform the same procedure on their other breast under adequate supervision to learn about regular practice of self-exam. They were recommended to perform monthly BSE to identify any changes in their breast, and in case of any unusual signs, immediately report to their healthcare providers. However, the typical changes in breast tissue, during pregnancy and mensuration cycle were comprehended. During the visit in the BAC women were asked some questions to assess their knowledge about breast screening, BSE and practicing it. Reponses from patients were recorded and analyzed. Women were also informed about recommendations for breast cancer screening for Pakistani female population in their native language.

### Recommendations for Screening Breast Cancer for Pakistani Women:

These recommendations have been extracted from three main sources viz: communication with expert oncologists from Pakistan, World Health Organization, and Centers of Diseases Control USA.

1. After the age of 20, every female should self-examine their breast after each menstrual cycle. Those females who are either pregnant or have reached menopause should set any specific date each month for BSE.
2. Females of the age group 20 to 39, after every three years, and females of age 40 or above, once a year, should get clinical breast examination by any surgeon or oncologist.
3. After the age of 40, mammogram of breasts should be done each year and women should carefully keep record of annual examinations.
4. Women with familial history of breast and/or ovarian cancer, early age incidence of BC in relatives or self, carriers of *BRCA I* or *BRCA II* mutations or close relatives of carriers and/or condition resulting in increased exposure to X-Rays before the age of 30 are considered being high risk individuals from any population. They must follow the recommendations more vigorously in order to avoid morbidity related to BC.
5. Clinicians should determine the age to stop screening with mammography after assessing each woman’s health status of-just advanced-age. According to the recent recommendations provided by CDC, women should discontinue screening mammograms at the age of 75 years.^14^

## RESULTS

**Fig.1:**
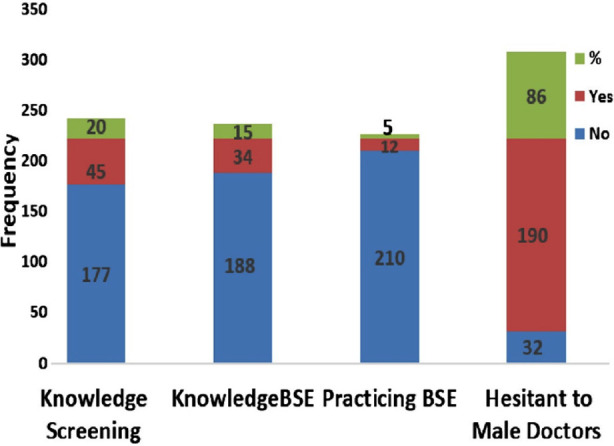
Frequency distribution of response recorded during Breast Awareness Clinics.

During the present study, 238 women visited BAC were found to have mean age of 29.97±8.9 years. Out of the total 222 valid recorded responses, 41% of the women reported that they had knowledge that breast cancer is the major threat to their health in Pakistan. Only 20% were aware of breast cancer screening but exhibited poor familiarity about the recommended program for Pakistani women. Knowledge about BSE was recorded only in 15% women, which was practiced regularly by only 12 out of total 222 women, however, this is 35.2% (12/34) of those who had knowledge about BSE. The last query was to assess hesitation to get screened by male doctor/health care provider. Only 14% women responded that for their health benefit they would not be hesitant in visiting male doctor for breast clinical examination (Fig.1). The utmost outcome of these clinics was that, three cases of most suspected malignancies were referred to specialist consultants and few cases were recommended for immediate mammograms.

## DISCUSSION

During the present study authors explored that 35.2% (12/34) of those who had knowledge about BSE are also used to practice it. However, those who used to practiced BSE make a small fraction of (5.4%) studied population, belonging to a small town of Chenab Nagar and surroundings. Similarly, a study carried out on female patients and attendants, visiting outdoor in Mayo Hospital Lahore, reported that 87.9% of them never practiced BSE.^15^ Another investigation showed that 26% of a group of 18-25 years old ladies from Dow medical college Karachi were practicing BSE.^16^ A survey conducted on 1000 students of different colleges of Karachi revealed that 71.4% knew about BSE while 33.1% of respondents reported a regular practice.^17^ Rasool et al 2019^18^ explored that 78% of the participants of the study had enough knowledge of BSE and 43.8% had knowledge about method to perform it but around 30% of those having knowledge about practice, used to perform BSE. A KAP study in two public hospitals of Karachi reported that most of the respondents (67.3%) were aware of BSE out of which around 31.9% regularly practice it too.^19^ The women who visited Holy Family hospital Rawalpindi, Pakistan reported that, 28.3% of them used to practice BSE.^20^ A recent study explored that even around 50% of medical professionals, nurses and senior medical students had misconceptions about BSE, these authors emphasized the need of training medical professionals especially nurses for active participation in breast care clinics.^21^ BSE was primarily anticipated as a widely accessible, non-invasive and inexpensive means to prevent presentation of late-stage breast cancerous lumps. However due to lack of evidence about the benefits of this practice in reducing mortality, international organizations began to suggest that BSE programs may prove to be beneficial for those populations which have lack of resources.^22^ Despite many investigators are refuting the effectiveness of BSE, numerous others proponents and BC awareness campaigners continue to reassure this practice. Some authors suggested that modality of BSE should be encouraged to as awareness plans rather than an effort for decrease in mortality.^23^,^24^ Indeed, screening by using mammography has well established role in reduction of mortality, however, its cost might not be affordable for women belongs to low- and middle-income countries. Thus, BSE still remained a beneficial method of screening and in case of any suspected finding followed by specialist consultation seems to be the best screening strategy for the low resources countries.^25^,^26^

The most communal cause about the delay in diagnosis is dearth of awareness about the significance of changes in the breast. Very low rates of early presentation have been found in Pakistani population mostly because of association with poor knowledge about the disease and its symptoms.^27^ A study was recently conducted in two main cancer hospitals of Karachi, Pakistan where, a mean delay of 15.7 ± 25.9 months, in seeking proper health consultation was reported.^28^ In this situation there is need to elaborate the screening centers in Pakistan. Currently existing breast cancer screening centers are associated with either the cancer Hospitals or General Hospitals in major cities. Thus, as reported by others, there is a deficiency of screening centers in Pakistan to facilitate early detection and diagnosis.^29^ While exploring reasons of delay in diagnosis of breast carcinomas in Pakistan investigators also found that 32.6% delays were attributed to the lack of accessibility of medical care facility.^30^ Many other findings confirmed that in the developing countries, lack of cognizance and familiarity of symptoms of breast malignancies along with the poor education level, and lack of accessibility of health centers are strongly associated with the delays of presentation of BC.^31^,^32^

Another significant finding of the current study was that 86% of the respondents were hesitant in visiting male doctors for BC screening. Thus, as Raza et al 2012^8^ suggested, increasing the number of trained female assistants with male GPs can help overcome this hurdle in regular screenings.

During a meta-analysis which comprised of eleven randomized research trials, 20% relative risk reduction of mortality has been recorded among the women who were invited for screening as compared to those women who were not screened.^19^ Another study reported that efficacy of BSE is a matter of contradiction thus, based upon large population surveys, authors concluded that there is not any significant association of practicing BSE in reduction of mortality.^20^ All research trials have not proved the effectiveness of BSE practice in reduction of mortality of breast carcinoma, despite the plentiful of literature accessible on BSE. However, women themselves can identify small lumps by BSE thus, contribute in overall healthier survival, by paving a way of confirmation by clinical diagnosis.^21^ Rivera and Rodriguez 2018^23^ suggested that modality of BSE should be encouraged as awareness plans rather than an effort for decrease in mortality (Rivera-Franco and Rodriguez, 2018^23^ and Thomas et al 2002^24^).

Owing to the lack of enough awareness, significant delay in diagnosis in Pakistani women, and dearth of screening centers, the authors want to float the idea of linking breast cancer screening with gynae clinics. Setting with Breast Awareness Clinic (BAC) in gynecology or health care centers in small towns or villages are more advisable with the involvement of female volunteer facilitators, for our hesitant female population to encourage regular screening and breast examinations. Moreover, this will help to make it easier for women to get themselves checked without specially taking out time for screening. The initiative of bringing together of gynecology setups and breast cancer screening centers can significantly increase the access of this facility to most of the female population in Pakistan.

### Limitations of present study:

Due to the COVID-19 restrictions, the breast awareness clinics had to be discontinued. As the data in current study is relatively small, it may have carried various biases and limitations. Despite the evident lacking in knowledge and regular practice of BSE, the studied population has generally positive attitude that provides a fertile ground for awareness dissemination campaigns. Relevant authorities can play an effective role by devising such cost-effective tools in preventive care of breast cancer in Pakistani women.

## CONCLUSION

The Healthcare providers always encourage breast self-examination but actual public awareness and training in the use of such imperative screening tool needs to be increased as the women in our population do not have a habit of actively taking care of their breast health. Suffice to say, as women become self-confident in performance of breast self-examination and plan for their regular annual clinical breast exams and mammograms, we will be able to bring about a positive change to reduce the currently devastating figures of breast cancer incidences and deaths in Pakistan in near future.

### Authors Contribution:

**DST:** Comprehended and designed the project & final approval of manuscript and responsible for integrity of the study.

**WT:** Reviewed the manuscript and suggested editions.

**BM:** Did data collection and contributed in manuscript editing.

**TU:** Contributed in manuscript writing.

## References

[1] Mubarik S, Malik SS, Wang Z, Li C, Fawad M, Yu C (2019). Recent insights into breast cancer incidence trends among four Asian countries using age-period-cohort model. Cancer Manag Res.

[2] Ghoncheh M, Pournamdar Z, Salehiniya H (2016). Incidence and mortality and epidemiology of breast cancer in the world. Asian Pac J Cancer Prev.

[3] Torre LA, Islami F, Siegel RL, Ward EM, Jemal A (2017). Global cancer in women:burden and trends. Cancer Epidemiol. Biomarkers Prev.

[4] (2020). GLOBOCAN:Global Cancer Statistics.

[5] Arshad S, ur Rehman M, Abid F, Yasir S, Qayyum M, Ashiq K (2019). Current situation of breast cancer in Pakistan with the available interventions. Int J Biosci.

[6] Gulzar F, Akhtar MS, Sadiq R, Bashir S, Jamil S, Baig SM (2019). Identifying the reasons for delayed presentation of Pakistani breast cancer patients at a tertiary care hospital. Cancer Manag Res.

[7] aNational Cancer Institute:Surveillance, Epidemiology and End Results Program:2021.

[8] Raza S, Sajun SZ, Selhorst CC (2012). Breast cancer in Pakistan:identifying local beliefs and knowledge. J. Am. Coll. Radiol.

[9] Bedi M, Devins GM (2016). Cultural considerations for South Asian women with breast cancer. J. Cancer Surviv.

[10] Saeed S, Asim M, Sohail MM (2021). Fears and barriers:problems in breast cancer diagnosis and treatment in Pakistan. BMC women's health.

[11] Gursoy AA, Hindistan S, Nural N, Kahriman I, Yilmaz F, Yigitbas Ç (2009). Comparison of three educational interventions on breast self-examination knowledge and health beliefs. Asian Pac J Cancer Prev.

[12] Al-Naggar RA, Al-Naggar DH, Bobryshev YV, Chen R, Assabri A (2011). Practice and barriers toward breast self-examination among young Malaysian women. Asian Pac J Cancer Prev.

[13] Erbil N, Nurgul B (2014). Health beliefs and breast self-examination among female university nursing students in Turkey. Asian Pac J Cancer Prev.

[14] Monticciolo DL, Newell MS, Moy L, Niell B, Monsees B, Sickles EA (2018). Breast cancer screening in women at higher-than-average risk:recommendations from the ACR. J Am Coll Radiol.

[15] Sarwar MZ, Shah SF, Yousaf MR, Ahmad QA, Khan SA (2015). Knowledge, attitude and practices amongst the Pakistani females towards breast cancer screening programme. J Pak Med Assoc.

[16] Memon ZA, Kanwal N, Sami M, Larik PA, Farooq MZ (2015). Risk of breast cancer among young women and importance of early screening. Asian Pac J Cancer Prev.

[17] Ahmed A, Zahid I, Ladiwala ZF, Sheikh R, Memon AS (2018). Breast self-examination awareness and practices in young women in developing countries:A survey of female students in Karachi, Pakistan. J. Educ. Health Promot.

[18] Rasool S, Iqbal M, Siddiqui A, Ahsan R, Mukhtar S, Naqvi S (2019). Knowledge, Attitude, Practice towards Breast Cancer and Breast Self-examination among Female Undergraduate Students in Karachi, Pakistan. J Adv Med Res.

[19] Ali A, Jameel N, Baig NN, SM ZH, SI AJ, Younus M (2020). Assessment of knowledge, attitude and practice regarding breast self-examination among females in Karachi. J Pak Med Assoc.

[20] Gilani SI, Khurram M, Mazhar T, Mir ST, Ali S, Tariq S (2010). Knowledge, attitude and practice of a Pakistani female cohort towards breast cancer. J Pak Med Assoc.

[21] Gul P, Mansoor M, Gul P, Arshad Z (2020). Breast cancer knowledge and perception among health care 3 professionals and senior medical students at Bolan Medical 4 Complex Hospital Quetta, Pakistan. J Pak Med Assoc.

[22] Pippin MM, Boyd R (2021). Breast Self-Examination. StatPearls [Internet]. 2020.

[23] Rivera-Franco MM, Leon-Rodriguez E (2018). Delays in breast cancer detection and treatment in developing countries. Breast cancer.

[24] Thomas DB, Gao DL, Ray RM, Wang WW, Allison CJ, Chen FL (2002). Randomized trial of breast self-examination in Shanghai:Final Results. J Natl Cancer Inst.

[25] Cazap E, Buzaid AC, Garbino C, de la Garza J, Orlandi FJ, Schwartsmann (2008). Breast cancer in Latin America:results of the SLACOM/BCRF expert survey. Cancer.

[26] Yip CH, Smith RA, Anderson BO, Miller AB, Thomas DB, Ang ES (2008). Guideline implementation for breast healthcare in low- and middle-income countries:early detection resource allocation. Cancer.

[27] Khan NH, Duan SF, Wu DD, Ji XY (2021). Better Reporting and Awareness Campaigns Needed for Breast Cancer in Pakistani Women. Cancer Manag Res.

[28] Shamsi U, Khan S, Azam I, Usman S, Maqbool A, Gill T (2020). Patient delay in breast cancer diagnosis in two hospitals in Karachi, Pakistan:Preventive and life-saving measures needed. J Glob Oncol.

[29] Menhas R, Umer S (2015). Breast cancer among Pakistani women. Iran J Public Health.

[30] Baig M, Sohail I, Altaf HN, Altaf OS (2019). Factors influencing delayed presentation of breast cancer at a tertiary care hospital in Pakistan. Cancer Rep.

[31] Maghous A, Rais F, Ahid S, Benhmidou N, Bellahamou K, Loughlimi H (2016). Factors influencing diagnosis delay of advanced breast cancer in Moroccan women. BMC Cancer.

[32] Ermiah E, Abdalla F, Buhmeida A, Larbesh E, Pyrhönen S, Collan Y (2012). Diagnosis delay in Libyan female breast cancer. BMC Res Notes.

